# Energetic supplementation for maintenance or development of *Apis mellifera* L. colonies

**DOI:** 10.1590/1678-9199-JVATITD-2020-0004

**Published:** 2020-05-15

**Authors:** Gabriela Pinto de Oliveira, Samir Moura Kadri, Bruno Giovane Emilio Benaglia, Paulo Eduardo Martins Ribolla, Ricardo de Oliveira Orsi

**Affiliations:** 1 Center for Education, Science and Technology in Rational Beekeeping (NECTAR), School of Veterinary Medicine and Animal Science (FMVZ), São Paulo State University (UNESP), Botucatu, SP, Brazil.; 2 Department of Parasitology, Botucatu Biosciences Institute (IBB), São Paulo State University (UNESP), Botucatu, SP, Brazil.

**Keywords:** Beekeeping, Apis mellifera, Energetic foods, Gene expression, Nutritional stress

## Abstract

**Background:**

The nutritional requirements of honeybees (*Apis mellifera*) for their complete development need to be supplied through food sources available in the environment, since honeybees are insects that depend directly on blossoming food sources. However, at certain times a food-supply reduction can promote nutritional stress, thus necessitating food supplementation for maintenance or production stimulus of the colonies. Thus, the determination of optimal energy supplementation can assist in the maintenance and production of colonies.

**Methods:**

Twenty *Apis mellifera* beehives were used (with five beehives per treatment): CTL, control (without feeding); SJ, sugarcane juice; SS, sugar syrup; and IS, inverted sucrose. We evaluated the food consumption, population development, and physiological state (expression of vitellogenin and hexamerin 70a genes) of each colony.

**Results:**

The results showed that the supplementation of colonies with sugar syrup resulted in an intermediate consumption level (894.6 ± 291 mL) and better development (384.9 ± 237.3 and 158.3 ± 171.6 cm^2^, sealed and open brood, respectively). Furthermore, this diet ensured that the colonies were in a good physiological state, as bees fed this diet presented the highest relative expression levels of vitellogenin and hexamerin 70a among all the diets tested.

**Conclusions:**

Therefore, sugar syrup is concluded to be the best artificial energetic food for use in the supplementation of honeybee colonies.

## Background

All of the nutritional needs of honeybees (*Apis mellifera*) for their complete development, maintenance, reproduction, production and longevity need to be supplied through food sources available in the environment [1]*.* In nature, *A. mellifera* bees meet all their nutritional needs through the ingestion of nectar and pollen [2]. Nectar has an undeniable importance to colony development, since it is the main source of energetic food for bees and permits their survival [3]. So, nutritional stress caused by food shortages or the availability of only foods of low nutritional value may lead to a reduction in the metabolic activity of the bees [4, 5]. It has also been observed that when there is little available natural food, there are reductions in the number of worker bees in the hive, queen’s egg-laying and survival rates of individuals, and increases in escape or abandonment rates [6], dramatically affecting the production of colonies.

Production of bee venom has been an increasingly profitable activity due to its use by the chemical and pharmaceutical industries. In addition, the venom is used for the production of antivenoms against Africanized honeybee stings [7]. Thus, the nutritional and physiological statuses of the colonies are important to bee venom production [7], given the possible occurrence of seasonal variations in the composition of the venoms [8]. As in the production of honey and other derivatives, colonies should be populous and well nourished to absorb the stress caused by the alarm pheromones released during venom harvest [8].

The nutritional stress can cause alterations in the bees’ metabolic pathways that influence several biological processes, including the expression of such genes as vitellogenin and hexamerin 70a. These genes are considered storage genes because they give rise to proteins that are produced during the larval stage and remain stored in the hemolymph and/or in the fat body [9].

Vitellogenin is important to the immune system and longevity of bees because this protein is a zinc carrier, and thus protects many cell lines against oxidative stress and apoptosis. Decreased expression of this gene product due to high expression levels of juvenile hormone was also previously shown to be related to reductions in the numbers of functional hemocytes in forager bees due to decreases in the quantity of zinc carried by vitellogenin [10, 11, 12, 13]. 

Hexamerin 70a is a gene related to storage, which is expressed in the larval, pupal, and adult stages of workers, queens, and drones, and is the only protein with a hexamerin subunit present in significant quantities in the fat body and adult hemolymph of *A. mellifera*. The synthesis of hexamerins, specifically the subunit 70a, like that of vitellogenin, is directly related to the quantity and quality of food intake. In older worker bees, such as forager bees, the levels of this protein are reduced due to the fact that such bees ingest a low-protein diet, since they do not collect and / or consume much pollen and instead preferentially consume nectar [9].

In a field experiment Carrillo [14] supplied *A. mellifera* colonies during an off-season with sugar syrup, sugarcane juice and inverted sucrose, and analyzed how it influences beeswax production. From this study they concluded that sugar syrup is directly correlated with an increase of beeswax production, showing that the source of sugar solution can directly influence the colony.

Beekeepers often provide artificial food to their colonies to lessen the negative effects that bee colonies suffer during this period in which food resources are drastically reduced, and thus ensure the survival and good performance of the colony [15]. Therefore, this study aimed to select the best energetic food to provide to bee colonies for optimizing their maintenance and growth. This was done while taking into account the food consumption, population development, and physiological state of the colony in relation to gene expression of vitellogenin and hexamerin 70a as a function of different foods provided to bee colonies.

## Methods

### Field experiment

For the field experiment, 20 *Apis mellifera* colonies in Langstroth beehives were selected, and the numbers of brood and food frames were standardized one month before the experiment with four brood, two food and two empty honeycomb frames. All experimental colonies were maintained in the apiary of the School of Veterinary Medicine and Animal Science (FMVZ), UNESP, on Lageado Experimental Farm, Botucatu, SP, Brazil. Queens were replaced in all colonies two months before the experiment. The food treatments used were the following: control (CTL), in which no artificial food was provided; feeding with sugarcane juice (SJ) produced in an electric cane mill at a research laboratory; feeding with sugar syrup (SS) prepared using pre-boiled filtered water and sucrose (50% sugar and 50% water, produced freshly on the same day it was offered to the colonies); and feeding with inverted sucrose (IS) purchased from Atrium Food Group, Campinas, São Paulo, Brazil (76 % sugar and 24% water).

Food was supplied twice a week in the amount 0.5 L per hive (1 L colony/per week) for a period of 60 days by means of a Boardman artificial feeder. The experiment was carried out in June and July of 2015. During the experimental period, food consumption was measured weekly.

Population development, including the numbers of open and sealed areas in one central nest structure, was measured monthly in the hives throughout the experimental period using the methods used and described by Ali [16]. The numbers of brood and food frames were quantified weekly, and were considered a brood or frame of food when 70% or more was occupied. Physicochemical analyses of the food provided were performed according to the following references. Total sugar reduction was performed according to Welke [17], calorimetric and dry matter analysis according to Sodré [18], and ash content according to Sodré [19].

### Honeybee collection

The bees for use in the analysis of the relative expression levels of the selected genes were collected on day 0, and were used as the experimental control. Collections were also carried out on day 30 and 60. Five worker bees were collected from the central frame, and were identified as intern bees (I); 5 worker bees were also collected that were carrying pollen in their corbicle, and in turn were identified as forager bees (F). During the experimental period, all of the colonies receiving the SJ treatment died, which made it impossible to collect these bees for analysis. After collection, the bees were immediately stored in a freezer at -80 °C for future RNA extraction.

### Relative expression of storage genes

To analyze the expression of vitellogenin and hexamerin 70a, 5 forager and 5 intern bees were randomly collected from each experimental colony and immediately frozen at -80 °C on days 0, 30, and 60. Total RNA was extracted from the heads of 5 bees of each type [20] using 500 μL of TRIzol® Reagent (Life Technologies) for each sample according to the manufacturer’s instructions. The extraction product was visualized on 1% agarose gel and quantified using a NanoDrop Instrument (ND-1000 Spectrophotometer). The RNA was treated with DNase I, incubated for 60 min at 37 °C and then for 10 min at 75 °C. Next, a solution was prepared of 0.75 mM oligo dT, 0.15 mM random oligos, 0.75 mM deoxynucleotide triphosphates and 1 μL of RNA, which was then incubated at 65 °C for 5 min and placed on ice for 1 min. We added 1× buffer dithiothreitol 0.005, RNaseOUT (40 U/μL), and 100 U SuperScript® III Reverse Transcriptase (Invitrogen) to this preparation. Complementary DNA synthesis was performed at 50 °C for 60 min, followed by 15 min at 70 °C.

Amplification was performed by real-time quantitative polymerase chain reaction (RT-qPCR) in a 25µL reaction mixture using the SYBR® Green PCR Master Mix (Applied Biosystems) and 0.2 μM of each primer. The sequences and details of the primers used are provided in Table 1. The RT-qPCR reactions were performed using ABI 7300 (Applied Biosystems) equipment under the following conditions: 1 cycle at 50 °C for 2 min; 1 cycle at 94 °C for 10 min; and 40 cycles of 94 °C for 15 s and 60 °C for 1 min. The dissociation curve was obtained under the following conditions: at 95 °C for 15 s, 60 °C for 30 s, and 95 °C for 15 s. The determination of the expression levels of vitellogenin and hexamerin 70a was performed in triplicate, and the expression of the actin gene was utilized as the control [20]. For each reaction, a negative control consisting of a mixture of reagents and water was also used.

**Table 1.** Oligonucleotides used in gene expression study of *Apis mellifera* that were fed different energetic foods during the off-season.

Gene	Accession number in Gene Bank	Sequence of primers 5’-3’	Amplified (pb)	Temperature^a^ (°C)	Efficiency of the oligonucleotides^b^ (%)
*Actin*	AB023025	TGCCAACACT GTCCTTTCTG AGAATTGACCCACC AATCCA	156	61	91,17
*Vitellogenin*	AJ517411	GCAGAATACA TGGACGGTGT GAACAGTCTTCGGA AGCTTG	146	61	110,17
*Hexamerin 70ª*	Martins et al. [9]	AAAGCCAATCACGCTCTGAT AATCGTGATTCAGATACCAGC	119	61	116

a Specific optimal annealing temperature for each primer.

b Measurement of the efficiency or real-time quantitative polymerase chain reaction (RT-PCR: calculated using the standard curve).

The efficiency of the oligonucleotides (E) was calculated from 4 dilutions of complementary DNA samples (1:5, 1:25, 1:125, and 1:625) using the formula E = 10 (-1/inclination). The quantification of a gene’s relative expression (R) was determined according to Pfaffl [21], defining the crossing point as the point at which the detected fluorescence was appreciably above the background fluorescence, using the formula:

$$R=\;\frac{E_{target}\times \;{\Delta CP}_{target}(Control\;-\;Sample)}{{\Delta CP}_{endogenous}(Control\;-\;Sample)\;\times \;E_{endogenous}}$$

### Statistical analyses

The data obtained for food consumption, population development, and gene expression were first tested for normality (Anderson-Darling test) and homogeneous variances (Levene’s test). When significant deviations (p < 0.05) from these assumptions were detected, the data were compared using the non-parametric Mann-Whitney test, and the median and interquartile intervals (Q1_Q3) were presented. When no significant deviations from normality or homoscedasticity were detected, the data were analyzed with one-way ANOVA, and the mean ± standard deviation values were presented. P-values ​​below 0.05 were considered significant. All statistical analyses were performed using the statistical software Minitab.

## Results

The respective weekly consumptions of SJ, SS, and IS were 0.994.8 ± 310, 894.6 ± 291, and 433.9 ± 227.6 mL. During the experimental period four colonies under SJ treatments absconded. 

The results of the physicochemical analyses of the different foods are presented in Table 2. Significant differences were observed in the analysis of SJ ash (0.27 ± 0.02%), whose value differed significantly from that of SS (0.01 ± 0.001%), but not from that of IS (0.11 ± 0.04%).

**Table 2.** Physicochemical analyses of different energetic foods (sugarcane juice, sugar syrup and inverted sucrose).

	Ash content (%)	Calorimetric (kcal kg^-1^)	Dry matter (%)	Reduced sugars (%)
**Sugarcane juice**	0.27 ± 0.02a	3,903	15.94 ± 0.00a	21.15 ± 1.6a
**Sugar syrup**	0.01 ± 0.00b	4,155	53.84 ± 0.41b	41.52 ± 2.8b
**Inverted sucrose**	0.11 ± 0.04ab	3,895	75.66 ± 0.75c	0.82 ± 0.0c

Different lowercase letters in the same column indicate statistical differences between means (Anderson-Darling test, p < 0.05).

The calorimetric analysis of the foods showed that SS presented the highest energetic value (4,155 kcal kg^-1^) of all the foods tested (3,903 kcal kg^-1^ for SJ and 3,895 kcal kg^-1^ for IS). The dry matter values of SJ and SS were lower than that of IS, indicating their higher moisture content. The analysis of the total reducing sugars in each food type found a higher SS value (41.52 ± 2.8%), which differed significantly from those detected for SJ (21.15 ± 1.6%) and IS (0.82 ± 0.01%). 

The data displayed in Table 3 represent the number of brood and food frames in colonies under different treatments. The number of brood frames was higher in colonies under the SS and IS treatments (3.60 ± 0.67 and 3 ± 1.10, respectively) compared to those in the CTL and SJ treatments (2.00 ± 0.90 and 1.90 ± 0.31, respectively). However, the number of food frames did not differ among treatments. The data shown in Table 4 represent the areas of open and sealed brood areas (cm²) observed in colonies subjected to different treatments. The treatments SS and IS presented larger sealed brood areas than the other treatments did. The largest of the open brood areas was recorded in the IS treatment. 

**Table 3.** Mean and standard deviation of the number of brood and food frames from control, sugarcane juice, sugar syrup and inverted sucrose treatments, throughout the experimental period.

Occupied frames in the nest with brood and food area				
**Frame**	**Control**	**Sugarcane juice**	**Sugar syrup**	**Inverted sucrose**
**Brood**	2.00 ± 0.90a	1.90 ± 0.31a	3.60 ± 0.67b	3.00 ± 1.10c
**Food**	4.52 ± 2.60a	3.84 ± 1.40a	4.00 ± 1.40a	4.32 ± 2.10a

Different lowercase letters on the same line indicate statistical differences between means (Anderson-Darling test, p < 0.05).

**Table 4.** Mean and standard deviation of the open and sealed (cm^2^) brood area in relation to the treatments control, sugarcane juice, sugar syrup and inverted sucrose, throughout the experimental period.

Population development (cm^2^)				
**Brood**	**Control**	**Sugarcane juice**	**Sugar syrup**	**Inverted sucrose**
**Sealed**	188.4 ± 132.2a	216.0 ± 167.5a	384.9±237.3b	401.7±194.0b
**Open**	82.9 ± 100.5a	100.9 ± 102.1ab	158.3±171.6ab	174.2±126.3b

Different lowercase letters on the same line indicate statistical differences between means (Anderson-Darling test, p < 0.05).

**Figure 1.**
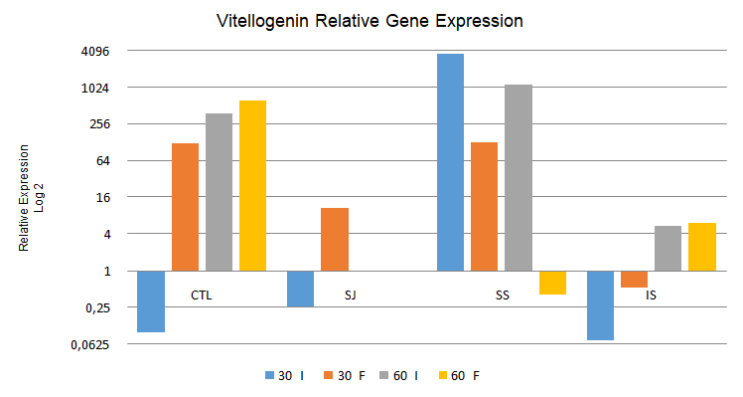
Relative expression of the vitellogenin gene in intern bees (I) and forager bees (F) under the different treatments used after 30 days and 60 days. CTL: control; SJ: sugarcane juice; SS: sugar syrup; IS: inverted sucrose. 60 I and 60 F: Data not obtained due to death of the colonies during the experimental period.

As to the relative expression of the vitellogenin gene, from the comparison between intern and forager bees at 30 and 60 days (Figure 1), the intern bees in the CTL, SJ, and IS treatments at 30 days and in the CTL and IS treatments at 60 days presented significantly lower relative expression levels compared to foragers at the same collection (p < 0.05). However, the contrary was observed in the SS treatment bees analyzed at both 30 and 60 days (p < 0.05), in which the intern bees expressed this gene at levels 2,862 times greater than those in the forager bees.

At 30 days, when analyzing only the intern bees in relation to the diets provided, the treatments CTL, SJ, and IS resulted in the downregulation of this gene (i.e., a decrease in the relative expression level of the gene in comparison to that in the control group), whereas in the SS treatment the upregulation of this gene (i.e. increased relative expression) was noted. Among the forager bees sampled at the same time, only those treated with IS presented downregulation of this gene, which showed that gene expression patterns differed between the intern and forager bees.

When analyzing intern bees at 60 days, the expression of this gene was upregulated in all treatment groups, although the CTL and SS treatments presented relatively higher expression levels compared to those in the IS treatment (67.92 and 205.63 times more than in the IS treatment, respectively). The bees fed SS showed greater expression of this gene in comparison to those in the other treatments, similarly to the intern bees collected at 30 days. However, among the forager bees collected at the same time, only those treated with SS presented downregulation of this gene in relation to the control.

When changing the focus of the data analysis and comparing the results obtained from intern and forager bees between 30 and 60 days under the different treatments to check for changes in the gene expression pattern after 60 days of feeding, it was observed that all the analyzed treatments differed in their relative expression levels (p < 0.05). In the CTL and IS treatments, both intern and forager bees showed increased expression of the vitellogenin gene during the experiment (P<0.05). However, the contrary occurred under the SS treatment, namely greater relative expression at 30 than at 60 days in both intern and forager bees.

**Figure 2.**
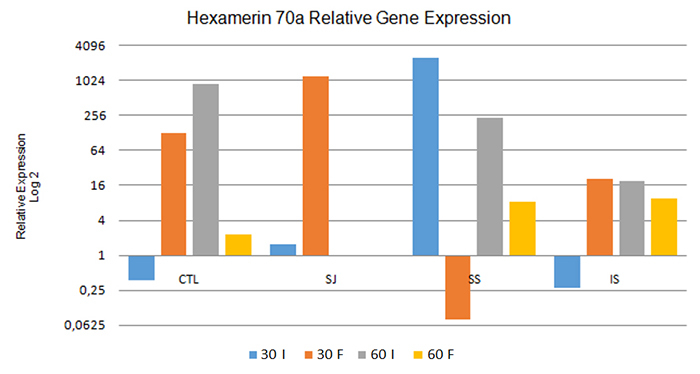
Relative expression of the hexamerin 70a gene in intern bees (I) and forager bees (F), after 30 days and 60 days, under the different treatments. CTL: control; SJ: sugarcane juice; SS: sugar syrup; IS: inverted sucrose. 60 I and 60 F: Data not obtained due to death of the colonies during the experimental period.

Comparing the intern and forager bees at 30 and 60 days, in all treatments except SS there were lower relative expression levels of the hexamerin 70a gene (Figure 2) in the intern bees than in the forager bees at 30 days, which indicates its downregulation in the intern bees and its upregulation in the forager bees. To the contrary, in the SS treatment the intern bees presented greater relative expression levels of hexamerin 70a than did the forager bees, while the intern bees displayed a very sharp upregulation. Specifically, the expression of this gene reached a value 33,483 times higher than that in intern bees at 30 days, whereas in the forager bees this gene’s expression was downregulated.

At 60 days, this gene presented upregulation in both the forager and intern bees in all treatments, but the intern bees showed relatively higher expression levels of the hexamerin 70a gene than the forager bees in all treatments.

## Discussion

Feeding artificial energetic foods to honeybee colonies during the off-season ensures the correct annual operation of the colony. For this to be effective, it is necessary to choose the best energetic food to offer the bees to ensure the proper development of a colony for the beekeeper. The lower consumption of inverted sucrose in the present study can be linked to the higher dry matter percentage and lower water content (24% water), since bees collect nectar as a natural energetic source whose water content exceeds 24% [22].

The dry matter data followed contrary trends among treatments in relation to the different food types, since the moisture content of the food is inversely proportional to dry matter [23, 24]. Therefore, sugar syrup and sugarcane juice contained more moisture, which may have favored their consumption since nectar, a natural energetic food of bees, has high humidity [25, 26]. Furthermore, the greater reduction of sugar and caloric content and the lower ash content of sugar syrup detected in the analyses carried out in this study, along with this food’s adequate dry matter content, indicated that this was the food that we supplied to the bees that most closely resembled honey, which is the main natural source of energy reserves for bee colonies. Thus, because it has a composition closer to that of the bees’ natural food, it was, at least in bromatological terms, the best source of artificial food for bees that was tested in this study.

Castagnino [27] showed that energetic supplementation during the off-season increases the queen’s egg-laying. In addition to supplying an energetic diet, a protein diet is also essential for colony maintenance and improving the queen’s egg-laying [28]. However, under the conditions of this experiment, the colonies had bee-bread reserves, and thus no protein supplementation was required. In this case, the energetic supplementation provided assisted in the maintenance of the colonies, and was able to account for the greater number of brood frames observed in the SS and IS treatments, suggesting that these energetic foods provided the necessary energetic support for the queen’s egg-laying during this period. Castagnino [27] obtained a large brood area in colonies fed sugar syrup, which was similar to the results of the present study. The energy provided by the consumption of the sugar syrup and inverted sucrose probably stimulated the queen’s egg-laying.

The loss of four colonies subjected to the SJ treatment over the experimental period probably occurred because the sugarcane juice (the diet offered to bees in the SJ treatment) may have undergone fermentation at ambient temperature [29]. Given this, it was not possible to obtain data on the relative expression of the tested genes at 60 days in the intern and forager bees in this treatment.

The analysis of intern bees at 30 days demonstrated an upregulation in vitellogenin expression in the SS treatment only. At the same moment and treatment the hexamerin 70a relative expression obtained a much greater value when compared with other treatments. This shows that sugar syrup had a more direct influence on intern bees than the other foods provided throughout the experimental period. This result may be related to several factors, such as energetic value and the maintenance of food integrity and quality at room temperature.

The vitellogenin expression levels observed after 60 days of feeding suggested that the SS treatment had a greater influence on the expression of this gene than the other diets, and it also facilitated better population development of the colony since the values of almost all of the performance parameters assessed were higher in this treatment compared to those in the other treatments. The forager bees, which live for approximately 21 days, presented less relative expression of this gene than the intern bees, which were less than 15 days old, at both 30 and 60 days. This may have been due to the fact that there was a higher concentration of juvenile hormone in the hemolymph of the older bees, which may have influenced their biosynthesis of vitellogenin. As noted earlier, vitellogenin is a protein related to the prevention of oxidative stress since it is a zinc carrier, while low levels of this protein can compromise the immune system [30].

## Conclusion

The results of this study demonstrated that the supplementation of honeybee colonies in the field during the off-season with sugar syrup results in an intermediate level of consumption of this food by them and greater colony development, and also ensured that the bees were in a better physiological state. Therefore, it is demonstrated that sugar syrup is the most beneficial artificial energetic food tested in this study.

### Abbreviations

CTL: control; F: forager bees; I: intern bees; S: inverted sucrose; RT-qPCR: real-time quantitative polymerase chain reaction; SJ: sugarcane juice; SS: sugar syrup.
